# Transcriptome analysis of FOXO-dependent hypoxia gene expression identifies Hipk as a regulator of low oxygen tolerance in *Drosophila*

**DOI:** 10.1093/g3journal/jkac263

**Published:** 2022-10-06

**Authors:** Kate Ding, Elizabeth C Barretto, Michael Johnston, Byoungchun Lee, Marco Gallo, Savraj S Grewal

**Affiliations:** Clark H. Smith Brain Tumour Centre, Arnie Charbonneau Cancer Institute, Alberta Children’s Hospital Research Institute, University of Calgary, Calgary, AB T2N 4N1, Canada; Department of Biochemistry and Molecular Biology Calgary, University of Calgary, Calgary, AB T2N 4N1, Canada; Clark H. Smith Brain Tumour Centre, Arnie Charbonneau Cancer Institute, Alberta Children’s Hospital Research Institute, University of Calgary, Calgary, AB T2N 4N1, Canada; Department of Biochemistry and Molecular Biology Calgary, University of Calgary, Calgary, AB T2N 4N1, Canada; Clark H. Smith Brain Tumour Centre, Arnie Charbonneau Cancer Institute, Alberta Children’s Hospital Research Institute, University of Calgary, Calgary, AB T2N 4N1, Canada; Department of Biochemistry and Molecular Biology Calgary, University of Calgary, Calgary, AB T2N 4N1, Canada; Clark H. Smith Brain Tumour Centre, Arnie Charbonneau Cancer Institute, Alberta Children’s Hospital Research Institute, University of Calgary, Calgary, AB T2N 4N1, Canada; Department of Biochemistry and Molecular Biology Calgary, University of Calgary, Calgary, AB T2N 4N1, Canada; Clark H. Smith Brain Tumour Centre, Arnie Charbonneau Cancer Institute, Alberta Children’s Hospital Research Institute, University of Calgary, Calgary, AB T2N 4N1, Canada; Department of Biochemistry and Molecular Biology Calgary, University of Calgary, Calgary, AB T2N 4N1, Canada; Clark H. Smith Brain Tumour Centre, Arnie Charbonneau Cancer Institute, Alberta Children’s Hospital Research Institute, University of Calgary, Calgary, AB T2N 4N1, Canada; Department of Biochemistry and Molecular Biology Calgary, University of Calgary, Calgary, AB T2N 4N1, Canada

**Keywords:** *Drosophila*, transcriptome, hypoxia, FOXO, Hipk, kinase, Hippo pathway, transcription, ribosome, mitochondria

## Abstract

When exposed to low oxygen or hypoxia, animals must alter their metabolism and physiology to ensure proper cell-, tissue-, and whole-body level adaptations to their hypoxic environment. These alterations often involve changes in gene expression. While extensive work has emphasized the importance of the HIF-1 alpha transcription factor on controlling hypoxia gene expression, less is known about other transcriptional mechanisms. We previously identified the transcription factor FOXO as a regulator of hypoxia tolerance in *Drosophila* larvae and adults. Here, we use an RNA-sequencing approach to identify FOXO-dependent changes in gene expression that are associated with these tolerance effects. We found that hypoxia altered the expression of over 2,000 genes and that ∼40% of these gene expression changes required FOXO. We discovered that hypoxia exposure led to a FOXO-dependent increase in genes involved in cell signaling, such as kinases, GTPase regulators, and regulators of the Hippo/Yorkie pathway. Among these, we identified homeodomain-interacting protein kinase as being required for hypoxia survival. We also found that hypoxia suppresses the expression of genes involved in ribosome synthesis and egg production, and we showed that hypoxia suppresses tRNA synthesis and mRNA translation and reduces female fecundity. Among the downregulated genes, we discovered that FOXO was required for the suppression of many ribosomal protein genes and genes involved in oxidative phosphorylation, pointing to a role for FOXO in limiting energetically costly processes such as protein synthesis and mitochondrial activity upon hypoxic stress. This work uncovers a widespread role for FOXO in mediating hypoxia changes in gene expression.

## Introduction

Animals often live in conditions where environmental oxygen levels fluctuate (Clegg 1997; Danovaro *et al.* 2010; Park *et al.* 2017). As a result, they must coordinate their physiology and metabolism with changes in oxygen availability to maintain proper homeostasis. This coordination can occur through alterations in gene expression and is essential for ensuring organismal survival in low oxygen (Bickler and Buck 2007; Ramirez *et al.* 2007; Harrison and Haddad 2011; Padilla and Ladage 2012; Nakazawa *et al.* 2016; Samanta *et al.* 2017; Harrison *et al.* 2018; Schito and Rey 2018; Holdsworth and Gibbs 2020).

Across all metazoans, perhaps the best-described and most intensively studied mechanism of gene regulation in hypoxia involves the HIF-1 alpha transcription factor (Semenza 2011, 2014b). When cells encounter low oxygen conditions, HIF-1 alpha protein is stabilized and translocates to the nucleus to induce gene expression. HIF-1 alpha-regulated genes encompass a diverse array of genes that are involved in biological processes such as metabolism, cell signaling and transcription, and that together coordinate cell-, tissue-, and whole-body level adaptations to low oxygen (Semenza 2011; Samanta *et al.* 2017). Studies in model organisms have identified how HIF-1 alpha is a key regulator of hypoxia in both normal physiology and in pathological disease states (Semenza 2014a, 2014b). However, compared with our understanding of HIF-1 alpha biology, less is known about other transcriptional mechanisms that contribute to both cellular and systemic oxygen homeostasis.

*Drosophila* have provided a versatile and informative model system for investigating organismal responses to hypoxia. In their natural ecology, *Drosophila* live and grow on rotting, fermenting food rich in microorganisms—an environment characterized by low ambient oxygen (Callier *et al.* 2015; Markow 2015; Harrison *et al.* 2018). They have therefore evolved mechanisms to tolerate hypoxia. For example, larvae and adults can tolerate severe hypoxia (∼1% oxygen) for up to 24 h with little impact on viability (Barretto *et al.* 2020), while embryos can survive complete anoxia (0% oxygen) for several days (Foe and Alberts 1985; Teodoro and O'Farrell 2003). Genetic studies have shown that flies can survive oxygen deprivation by increasing tracheal branching to expand oxygen supply to tissues (Centanin *et al.* 2008; Wong *et al.* 2014), and by remodeling their physiology and metabolism through both HIF-1 alpha-dependent and independent mechanisms (Wingrove and O'Farrell 1999; Lavista-Llanos *et al.* 2002; Teodoro and O'Farrell 2003; Centanin *et al.* 2005; Romero *et al.* 2007; Harrison and Haddad 2011; Morton 2011; Li *et al.* 2013; Bandarra *et al.* 2014; Harrison *et al.* 2018; Lee *et al.* 2019; Texada *et al.* 2019; Barretto *et al.* 2020). Relatively few studies, however, have used genome-wide approaches to identify gene expression changes associated with adaptation to hypoxia in *Drosophila* (Liu, Roy, *et al.* 2006; Li *et al.* 2013). One study examined transcriptome changes associated with larval hypoxia and identified widespread changes in metabolic gene expression (Li *et al.* 2013). This study also showed that of the hundreds of gene expression changes, over half were independent of HIF-1 alpha, emphasizing the importance of additional transcriptional mechanisms in the control of hypoxia gene expression (Li *et al.* 2013).

Using *Drosophila* larvae and adults, we previously identified the transcription factor, Forkhead Box O (FOXO), as a regulator of hypoxia tolerance (Barretto *et al.* 2020). FOXO is a conserved regulator of stress responses and animal aging (Webb and Brunet 2014). Studies in *Drosophila* have shown it is induced by stressors such as starvation, oxidative stress, pathogens, and ionizing radiation (Jünger *et al.* 2003; Dionne *et al.* 2006; Karpac *et al.* 2009; Karpac *et al.* 2011; Borch Jensen *et al.* 2017). Genetic studies have also shown that, in general, loss of *foxo* induces stress sensitivity and shortens lifespan whereas increased FOXO activity, particularly in tissues such as gut, muscle, and fat body can promote stress resistance and extend lifespan (Giannakou *et al.* 2004; Hwangbo *et al.* 2004; Tettweiler *et al.* 2005; Kramer *et al.* 2008; Demontis and Perrimon 2010; Alic, Giannakou, *et al.* 2014; Alic, Tullet, *et al.* 2014; Dobson *et al.* 2017). We showed that FOXO activity is rapidly induced in hypoxia and that it is needed for hypoxia survival in both larvae and adults. We also identified the immune Relish/NF Kappa B transcription factor as one target of FOXO important for its hypoxia tolerance effects. However, it is unclear what other genes FOXO may regulate in hypoxia. Previous studies have shown that under normal conditions, FOXO can bind to thousands of genomic loci (Alic *et al.* 2011; Birnbaum *et al.* 2019) and can regulate the expression of hundreds genes in a tissue- and context-specific manner (Alic *et al.* 2011; Alic, Giannakou, *et al.* 2014; Alic, Tullet, *et al.* 2014), raising the possibility that it may mediate broad effects on gene expression in hypoxia.

In this report we describe our transcriptome analysis of hypoxia-mediated gene expression changes upon hypoxia in adult flies. We show that FOXO is required for upregulation of genes involved in cell signaling and we identify the kinase Hipk as a regulator of hypoxia tolerance. We also see that FOXO suppresses expression of genes involved in protein synthesis and mitochondrial activity, suggesting it plays an important role in limiting energetically costly processes in low oxygen stress.

## Materials and methods

### *Drosophila* stocks and culturing

Flies were grown on medium containing 150 g of agar, 1,600 g of cornmeal, 770 g of Torula yeast, 675 g of sucrose, 2,340 g of d-glucose, 240 ml of acid mixture (propionic acid/phosphoric acid) per 34 l of water. All stocks were maintained at either 18°C or room temperature. For adult hypoxia exposures, flies were raised from embryos to adults at 25°C and then, following eclosion, females were allowed to mate for 2 days before being separated from males and aged for another 5–6 days, at which time point hypoxia experiments were performed. For larval hypoxia exposures, hatched larvae were grown on food at 25°C until 96 h after egg-laying, at which time point hypoxia experiments were performed. The following *Drosophila* strains were used: *w^1118^, foxo^Δ94^/TM6B* (Slack *et al.* 2011), *UAS-hipk* RNAi (Bloomington Drosophila Stock Centre # 35363), *da-GS-Gal4 (daughterless-GeneSwitch)* (Sun *et al.* 2014). For UAS gene induction using the GeneSwitch system, adult flies were fed food supplemented with RU486 (200 µM) for 7 days. Control (noninduced flies) were maintained on normal food supplemented with ethanol (vehicle control for RU486). We found that RU486 treatment had no effect on hypoxia survival (Supplementary Fig. 2) making it a useful system for examining the effects of adult stage-restricted genetic manipulation on hypoxia tolerance.

### Hypoxia exposure and measurement of hypoxia survival

Vials of adult flies or larvae were placed into an airtight glass chamber into which a mix of 1% oxygen/99% nitrogen gas continually flowed. Flow rate was controlled using an Aalborg model P gas flow meter. Normoxic animals were maintained in vials in ambient air. For hypoxia survival experiments, mated female adults were placed in placed into hypoxia (1% oxygen) for 20 h in groups of 15 flies per vial. Then, vials were removed from hypoxia and the flies were allowed to recover before the numbers of dead flies were counted.

### Total RNA isolation

Adult flies (5 per group), adult tissues (from 10 animals per group), or larvae (10 per group) were snap frozen on dry ice. Total RNA was then isolated using Trizol according to the manufacturer’s instructions (Invitrogen; 15596-018). Extracted RNA was then DNase treated (Ambion; 2238G) to be used for subsequent qPCR or mRNA-sequencing.

### mRNA-sequencing and RNA-seq analyses

Three to four independent biological replicates (5 flies per group) of normoxia- and hypoxia-exposed groups of *w^1118^* and *foxo* mutants were prepared and analyzed. RNA-sequencing was conducted by the University of Calgary Centre for Health Genomics and Informatics. The RNA Integrity Number (RIN) was determined for each RNA sample. Samples with an RIN score higher than 8 were considered good quality, and Poly-A mRNA-seq libraries from such samples were prepared using the Ultra II Directional RNA Library kit (New England BioLabs) according to the manufacturer’s instructions. Libraries were then quantified using the Kapa qPCR Library Quantitation kit (Roche) according to the manufacturer’s directions. Finally, RNA libraries were sequenced for 100 cycles using the NextSeq 500 Sequencing System (Illumina). Transcripts were quantified using kallisto (Bray *et al.* 2016) referencing refSeq mRNA (release: October 15, 2019) corresponding to dm6 annotation. Differential expression testing was performed using sleuth (Pimentel *et al.* 2017).

### Gene Ontology, KEGG pathway, and tissue expression analyses

Analyses of Gene Ontology and KEGG pathway enrichment of up- and downregulated genes [>1.5-fold, *q*-val (FDR-corrected *P*-val) <0.05] were performed using G-profiler (Raudvere *et al.* 2019) and Revigo (Supek *et al.* 2011).

### Quantitative RT-PCR measurements

Total RNA was extracted from either whole flies, whole larvae, or isolated adult tissues. The RNA was then DNase treated as described above and reverse transcribed using Superscript II (Invitrogen; 100004925). The generated cDNA was used as a template to perform qRT-PCRs (ABI 7500 real time PCR system using SyBr Green PCR mix) using gene-specific primers. PCR data were normalized to *beta tubulin* or *eIF2 alpha* mRNA levels. The following primers were used:

*tRNA ala* forward: GCGGCCGCACTTTCACTGACCGGAAACG

*tRNA ala* reverse: GCGGCCGCGCCCGTTCTAACTTTTTGGA

*tRNA arg* forward: GCGGCCGCGTCCGTCCACCAATGAA AAT

*tRNA arg* reverse: GCGGCCGCCGGCTAGCTCAGTCGGT AGA

*tRNA eMet* forward: GCGGCCGCCGTGGCAATCTTCTGAA ACC

*tRNA eMet* reverse: GCGGCCGCTCAGTGGAAAACCATA TGTTCG

*tRNA iMet* forward: AGAGTGGCGCAGTGGAAG

*tRNA iMet* reverse: AGAGCAAGGTTTCGATCCTC

*beta tubulin* forward: ATCATCACACACGGACAGG

*beta tubulin* reverse: GAGCTGGATGATGGGGAGTA

*Hipk* forward: CAACAATGTCAAGGCATC

*Hipk* reverse: CAGGCTGCACAGTGTGGAAA

*eIF2 alpha* forward: TCTTCGATGAGTGCAACCTG

*eIF2 alpha* reverse: CCTCGTAACCGTAGCAGGAG

### Polysome profiling

Larvae were lysed in lysis buffer [25 mM Tris, pH 7.4, 10 mM MgCl_2_, 250 mM NaCl, 1% Triton X-100, 0.5% sodium deoxycholate, 0.5 mM DTT, 100 mg/ml cycloheximide, 1 mg/ml heparin, 16 complete mini roche protease inhibitor, 2.5 mM PMSF, 5 mM sodium fluoride, 1 mM sodium orthovanadate and 200 U/ml ribolock RNAse inhibitor (Fermentas)] using a Dounce homogenizer. Lysates were then centrifuged at 15,000 rpm for 20 min and the supernatant was removed carefully using a fine syringe to avoid the floating fat content. For each condition, lysates containing 300 mg of total RNA were then layered on top of a 15–45% w/w sucrose gradient (made using 25 mM Tris, pH 7.4, 10 mM MgCl_2_, 250 mM NaCl, 1 mg/ml heparin, 100 mg/ml cycloheximide in 12 ml polyallomer tube) and centrifuged at 37,000 rpm for 150 min in a Beckmann Coulter Optima L-90K ultracentrifuge using a SW-41 rotor. Polysome profiles were obtained by pushing the gradient using 70% w/v sucrose pumped at 1.5 ml/min into a continuous OD254 nm reader (ISCO UA6 UV detector).

### Fecundity assay

One- to two-day-old virgin *w^1118^* females were allowed to mate with males for 2 days and then the females were separated and either maintained in normoxia (controls) or exposed to hypoxia for either 8 or 12 h before being returned to normoxia. Twenty-four hours later, the females were transferred in groups of 3 to new vials and allowed to lay eggs for a 24-h period (day 1), and then transferred to a second set of vials to lay eggs for a further 24-h period (day 2). Fecundity was then assessed by measuring the number of viable pupae per female that emerged from eggs laid on days 1 and 2.

### Statistical analysis of qRT-PCR and fecundity data

Data were analyzed by Student’s *t*-test or 2-way ANOVA followed by post hoc tests where appropriate. All statistical analyses and data plots were performed using Prism statistical software. Differences were considered significant when *P*-values were <0.05.

## Results

### Hypoxia leads to upregulation of transcription factor and kinase gene expression

**Fig. 1.**
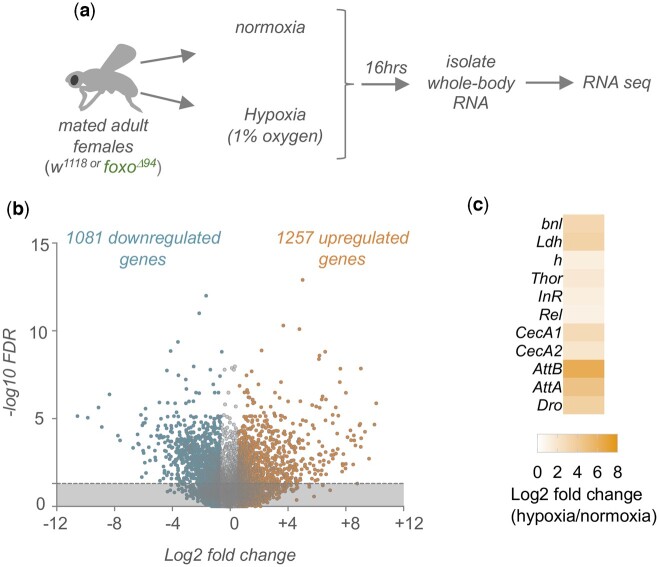
Hypoxia-induced alterations in whole-body gene expression. a) Schematic outline of our experimental approach. b) Volcano plot showing the up- (orange) and downregulated (blue) genes following hypoxia exposure. Genes were considered differentially expressed if they showed a significant [*q*-val (FDR-corrected *P*-val) <0.05] change in expression that was > ±1.5-fold different in hypoxia vs normoxia. Dashed line indicates *q*-val = 0.05. c) Heatmap depicting the change in expression (Log2-fold change, hypoxia vs normoxia conditions) of previously described hypoxia-induced genes.

Adult mated *w^1118^* (control) or *foxo^Δ94^* (foxo null mutant) (Slack *et al.* 2011) females were either maintained in normoxia or exposed to hypoxia (1% oxygen) for 16 h and we then isolated whole-body RNA for RNA-seq analysis (Fig. 1a). We first examined the gene expression changes induced by hypoxia in the control animals. Using a cutoff of ±1.5-fold and a false-discovery rate corrected *P*-value <0.05, we identified 1,081 genes with reduced mRNA expression and 1,257 genes with increased mRNA expression in *w^1118^* animals (Fig. 1b and Supplementary Table 1). Among the upregulated genes, we saw increased expression of several genes previously shown to be induced upon hypoxia exposure in larvae and/or adults. For example, we saw increased expression of the fly fibroblast growth factor homolog, *branchless (bnl)*, the glycolytic enzyme, *Lactate dehydrogenease (Ldh/ImpL3)*, and the transcriptional repressor, *hairy (h)*, each of which has been shown to be upregulated upon hypoxia exposure (Centanin *et al.* 2008; Zhou *et al.* 2008; Li *et al.* 2013) (Fig. 1c). We previously showed hypoxia induces rapid nuclear localization and increased transcriptional activity of the transcription factor FOXO, which we found promoted hypoxia tolerance by increasing expression of the innate immune transcription factor Relish (*Rel*) (Barretto *et al.* 2020). Consistent with this, our transcriptome data showed that hypoxia led to increased expression of two FOXO target genes, *Thor* and *InR*, and increased expression of *Rel* and antimicrobial peptide genes (e.g. *CecA1*, *CecA2*, *AttB*, *AttA*, *Dro*), which are known targets of Relish (Fig. 1c)*.* Together these changes in gene expression confirm that our low oxygen exposure protocol induced a robust hypoxic response. Two previous studies used genome-wide transcriptome analyses to examine hypoxia-regulated genes in *Drosophila* (Liu, Roy, *et al.* 2006; Li *et al.* 2013). Like us, Liu *et al.* examined hypoxia in adult female flies, and they used DNA microarray hybridization to identify genes that showed significantly increased expression after 6 h of severe (0.5% oxygen) hypoxia exposure. They identified 79 genes, of which 47 (59%) were also identified in our RNA-seq analysis (significant overlap, *P* = 7.3 × 10^−29^) (Supplementary Table 1). Li *et al.* also used DNA microarray hybridization to detect hypoxia-regulated genes, in this case, in late L3 larvae using milder hypoxia (4% oxygen). They identified 627 significantly (*P* < 0.01) upregulated (>1.5-fold) genes, of which 130 (21%) were also identified as upregulated in our RNA-seq analysis (significant overlap, *P* = 2.2 × 10^−19^), and they identified 417 significantly (*P* < 0.01) downregulated (>1.5-fold) genes, of which 80 (19%) were also identified in our RNA-seq analysis (significant overlap, *P* = 4.8 × 10^−13^) (Supplementary Table 1). Thus, even though this study analyzed hypoxia at a different stage of the life cycle and at a different concentration of oxygen, one-fifth of the genes that were identified as being hypoxia-regulated in larvae were also identified in our study in adults. The differences in the number of genes identified in our study *vs.* the previous studies likely reflect differences in biology between larvae and adults, and the greater sensitivity of RNA-seq approaches to detect differentially expressed genes compared with DNA microarrays.

**Fig. 2.**
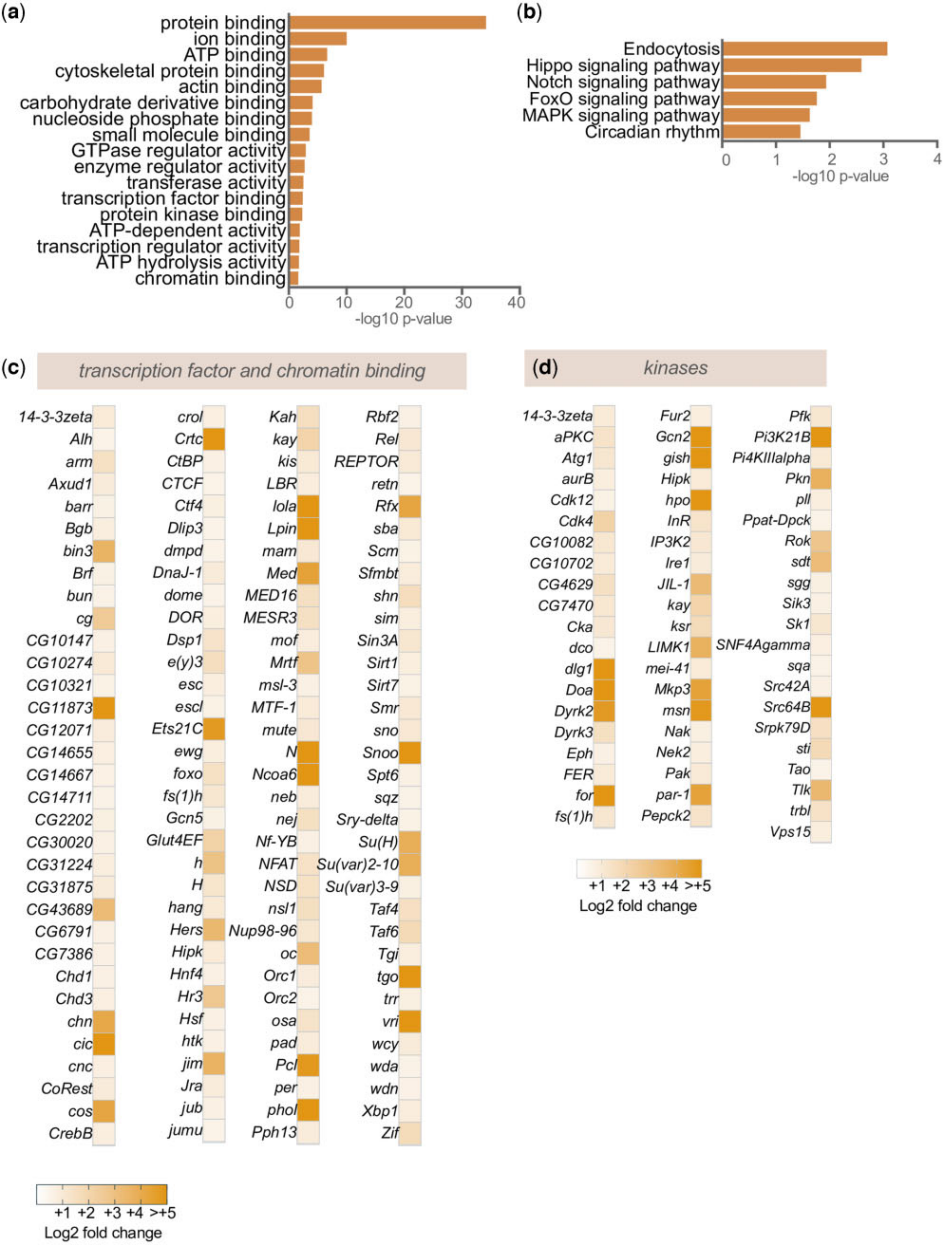
Hypoxia upregulates mRNA expression of transcription factors and kinase genes. a) GO analysis (molecular function category) and b) KEGG pathway analysis of genes showing >1.5-fold increase following hypoxia exposure. c) Heatmap depicting the increases in mRNA expression (Log2-fold change hypoxia vs normoxia) of transcription factor genes. d) Heatmap depicting the increases in mRNA expression (Log2-fold change hypoxia vs normoxia) of kinase genes.

We used Gene Ontology analysis to examine the genes that showed upregulated expression upon hypoxia. This analysis showed that the upregulated genes were particularly enriched for gene categories related to chromatin modification and transcription, small G-protein regulators, and kinases (Fig. 2a). In addition, KEGG pathway analysis of the upregulated genes showed enrichment for genes involved in Hippo, Notch, FOXO, and MAPK signaling (Fig. 2b). We saw hypoxia-induced increases in gene expression for 132 regulators of transcription and chromatin, and 61 kinases (Fig. 2, c and d). Together, these analyses suggest that hypoxia leads to widespread upregulation of different signaling pathways and transcriptional responses.

### FOXO is required for hypoxia-induced upregulation of signaling molecules and regulators of the Hippo pathway

**Fig. 3.**
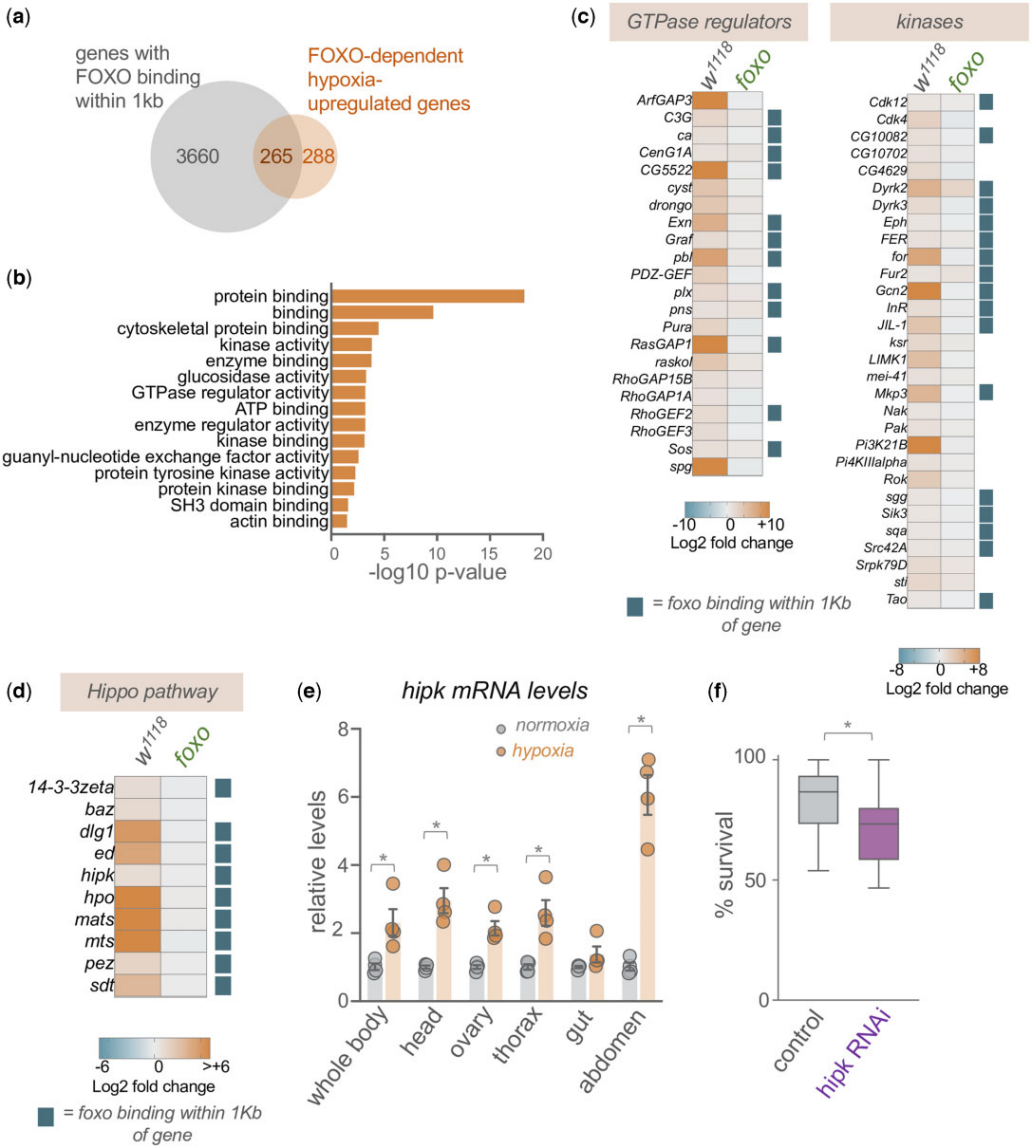
Hipk is a hypoxia-induced gene required for organismal hypoxia tolerance. a) Venn diagram showing overlap between genes previously shown to have FOXO binding within 1 kb, as detected by ChIP, and FOXO-dependent upregulated genes identified in the present study. b) GO analysis (molecular function category) of FOXO-dependent hypoxia-induced genes (genes showing a significant >1.5-fold increase in mRNA expression following hypoxia exposure in *w^1118^* but not *foxo* mutants). c) Heatmap depicting the increases in mRNA expression (Log2-fold change, hypoxia vs normoxia) of GTPase regulators and kinases in *w^1118^* and *foxo* mutants. Blue squares indicate genes previously shown to have FOXO binding within 1 kb of the gene as measured by ChIP. d) Heatmap depicting the increases in mRNA expression (Log2-fold change, hypoxia vs normoxia) of Hippo pathway genes in *w^1118^* and *foxo* mutants. Blue squares indicate genes previously shown to have FOXO binding within 1 kb of the gene as measured by ChIP. e) qPCR analysis of *hipk* mRNA levels from normoxia vs hypoxia exposed animals. RNA was isolated from either whole animals or specific tissues. Bars represent mean ± SEM. Symbols represent individual data points, *n* = 4 per condition. *P < 0.05, Student’s *t*-test. f) Hypoxia survival of control (*da-GSG>hipk RNAi*, no RU486) vs *hipk* RNAi (*da-GSG>Hipk RNAi*, RU486-treated) adult flies. Data are presented as box plots (25%, median and 75% values) with error bars indicating the min and max values, *n* = 14 groups of flies per condition.

To identify FOXO-dependent hypoxia-induced genes we identified genes that showed a significant (*q*-val <0.05), >1.5-fold upregulation upon hypoxia in *w^1118^* but not *foxo^Δ94^*. Using this criteria, we found that of the 1,257 genes that were upregulated upon hypoxia exposure, 551 (44%) were not significantly upregulated in *foxo* mutants, suggesting that a large proportion of hypoxia-induced gene expression requires FOXO activity (Supplementary Table 1). Two previous studies used ChIP-chip and ChIP-seq approaches to identify FOXO genomic binding sites in young female adult flies, and, between them, identified 3,925 loci that bound FOXO and were within 1 kb of a protein coding gene (Alic *et al.* 2011; Birnbaum *et al.* 2019). Interestingly, we saw that of the 553 FOXO-dependent hypoxia-upregulated genes that we identified, 265 (48%) overlapped with these FOXO-bound genes (Fig. 3a), suggesting that almost half the FOXO-dependent hypoxia genes may be induced by direct FOXO transcriptional activation (Supplementary Table 1). We used Gene Ontology analysis to examine the 553 FOXO-dependent hypoxia-upregulated genes. The main classes of genes identified were largely related to signaling regulators, such as kinases, GTPase regulators, and guanine-nucleotide exchange factors (Fig. 3, b and c). For example, we saw that many GTPase regulators required FOXO for their hypoxia upregulation and many of these contained FOXO-binding sites within 1 kb of their gene coding region (Fig. 3c). In addition, almost half the kinases that we saw were induced in hypoxia were dependent on FOXO for their induction (Fig. 3c). Interestingly, among these signaling molecules, we saw enrichment in regulators of the Hippo signaling pathway, many of which were previously shown to be enriched among FOXO-bound genes, suggesting that they may be directly regulated by FOXO (Fig. 3d).

### Hipk is upregulated in hypoxia and modulates hypoxia tolerance

One regulator of the Hippo pathway we saw upregulated and associated with a FOXO DNA binding site was Homeodomain interacting protein kinase (Hipk). In *Drosophila*, Hipk has been shown to control metabolism and growth in epithelial tissues, and has been shown to function as a regulator of several signaling pathways including Hippo, Wingless, Notch, JAK/STAT and JNK (Lee, Andrews, *et al.* 2009; Lee, Swarup, *et al.* 2009; Chen and Verheyen 2012; Poon *et al.* 2012; Verheyen *et al.* 2012; Blaquiere *et al.* 2014; Tettweiler *et al.* 2019; Wong *et al.* 2019; Kinsey *et al.* 2021; Steinmetz *et al.* 2021). In addition, a recent study in *C. elegans* showed that the worm homolog of Hipk, hpk1, was a regulator of worm survival in low oxygen (Doering *et al.* 2022). We therefore examined the role of Hipk in *Drosophila* hypoxia in more detail. Using qRT-PCR we confirmed that hypoxia exposure led to an increase in *hipk* mRNA levels in whole animals. We also saw hypoxia-induced increases in *hipk* mRNA levels in specific tissues such as the head, thorax (which is enriched in muscle), ovaries, and abdomen (which is enriched in adipose tissues), suggesting that the hypoxia-mediated increase in Hipk expression occurred across many tissues (Fig. 3e). To explore the functional role for Hipk in hypoxia, we used the RNAi to knockdown *hipk* in flies and examined the effects on hypoxia tolerance. We used the *daughterless-GeneSwitch-Gal4 (da-GSG)* driver to induce ubiquitous expression of the dsRNA and to restrict RNAi-mediated knockdown of *hipk* to adult stages. We fed *da-GSG>hipk RNAi* females either normal food (control) or RU486-containing food to induce RNAi (*hipk RNAi*), which we found induced ∼50% decrease in *hipk* mRNA levels (Supplementary Fig. 1), and then examined the effects on hypoxia survival. We found that following 20 h of hypoxia exposure the *hipk RNAi* animals had significantly reduced hypoxia survival compared with the control flies, suggesting that Hipk is required for hypoxia tolerance (Fig. 3f)

### Hypoxia downregulates expression of protein synthesis and egg production genes and leads to reduced translation and fecundity

**Fig. 4.**
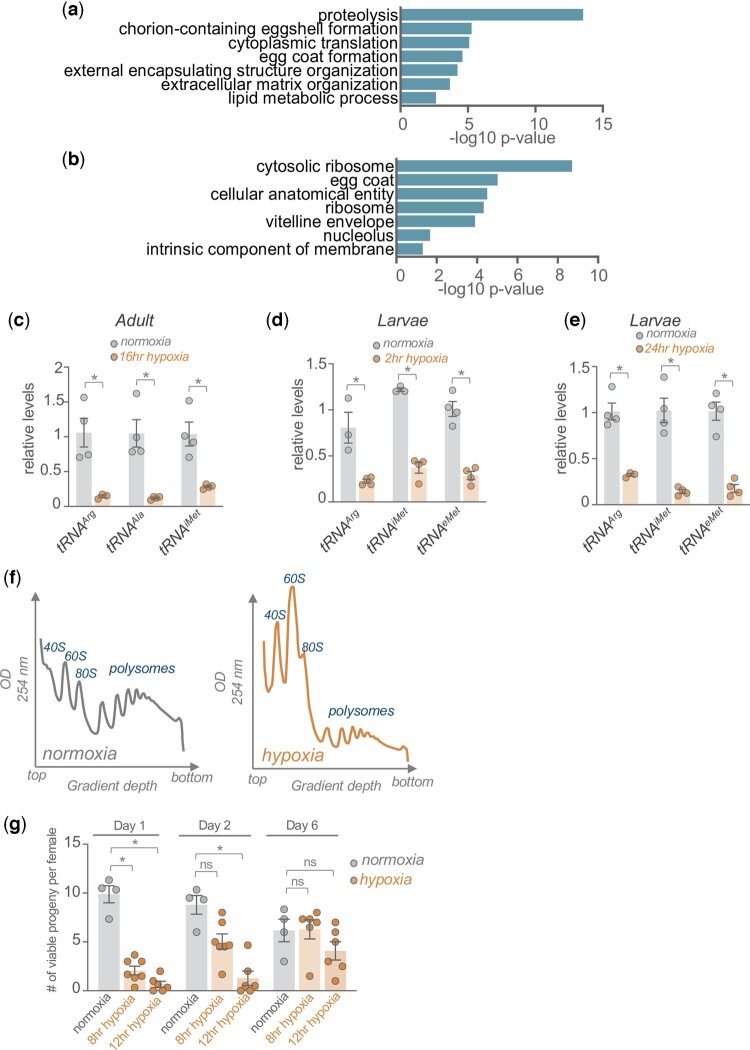
Hypoxia downregulates mRNA expression of protein synthesis and egg formation genes and leads to reduced translation and decreased fecundity. GO analysis (a, biological process category and b, cellular component category) of genes showing >1.5-fold decrease in expression following hypoxia exposure. qRT-PCR measurement of tRNA levels following (c), 2 h or (d), 24 h of hypoxia exposure in developing larvae. Bars represent mean ± SEM. Symbols represent individual data points, *n* = 4 per condition. *P < 0.05, Student’s *t*-test. f) Polysome profiles of normoxia (left) and hypoxia (right) exposed larvae. Plots indicate continuous OD 254 nm measurements from fractionated whole-body lysates. Peaks corresponding to 40S, 60S, 80S, and polysomes are indicated. The top and bottom lysate fractions from the centrifuged sucrose gradients are indicated. g) Fecundity measurements from mated females exposed to normoxia or 8 or 12 h of hypoxia. Data show the mean number of viable pupae per female that developed from eggs laid on day 1 or day 2 following the hypoxia exposure. Bars represent mean ± SEM. Symbols represent individual data points, *n* = 4 per condition. *P < 0.05, Student’s *t*-test following 2-way ANOVA.

We used Gene Ontology analysis to examine the genes that showed significantly reduced (>1.5-fold decrease) expression upon hypoxia in control *w^1118^* animals. We saw enrichment in genes involved in ribosome function, egg formation, and proteolysis (Fig. 4, a and b). Almost all the proteolysis genes were proteases that showed enriched expression in either the intestine or fat body (Supplementary Table 1). The decreased expression of genes involved in ribosome function is consistent with suppressed protein synthesis, a widely seen response to hypoxia in different organisms (Hofmann and Hand 1994; Hochachka *et al.* 1996; Liu, Cash, *et al.* 2006; van den Beucken *et al.* 2006; Anderson *et al.* 2009; Scott *et al.* 2013). We previously showed that regulation of tRNA synthesis was a key mechanism for regulating protein synthesis in *Drosophila*, particularly in response to nutrient starvation (Marshall *et al.* 2012; Rideout *et al.* 2012; Sriskanthadevan-Pirahas *et al.* 2018). When we examined tRNA levels by qPCR, we saw a strong reduction following hypoxia exposure (1% oxygen) in adults (Fig. 4c). Furthermore, we saw that exposure of larvae to 1% oxygen also led to a strong reduction in tRNA levels that was observed at both 2 and 24 h of hypoxia exposure (Fig. 4, d and e). We also saw that hypoxia larvae showed a similarly rapid decrease in overall translation compared with normoxic animals as shown by a decrease in polysome: monosome ratios in polysome profiles from whole animal lysates (Fig. 4f). These results indicate that global suppression of protein synthesis is a common response to extreme hypoxia in both larvae and adults.

Given the decreased expression of egg formation genes, we also examined whether hypoxia might impact female fecundity. To do this, we exposed mated *w^1118^* females to hypoxia for either 8 or 12 h, allowed them to recover for a day, and then measured how many viable progeny they produced in the subsequent 6 days. We saw that when exposed to either 8 or 12 h of hypoxia, females produced significantly fewer viable progeny at 1 and 2 days post-hypoxia compared with normoxic control females (Fig. 4g). These results indicate that a brief exposure to hypoxia can transiently suppress fecundity in female flies.

### FOXO is needed for hypoxia-mediated decreases in ribosome and mitochondrial gene expression

**Fig. 5.**
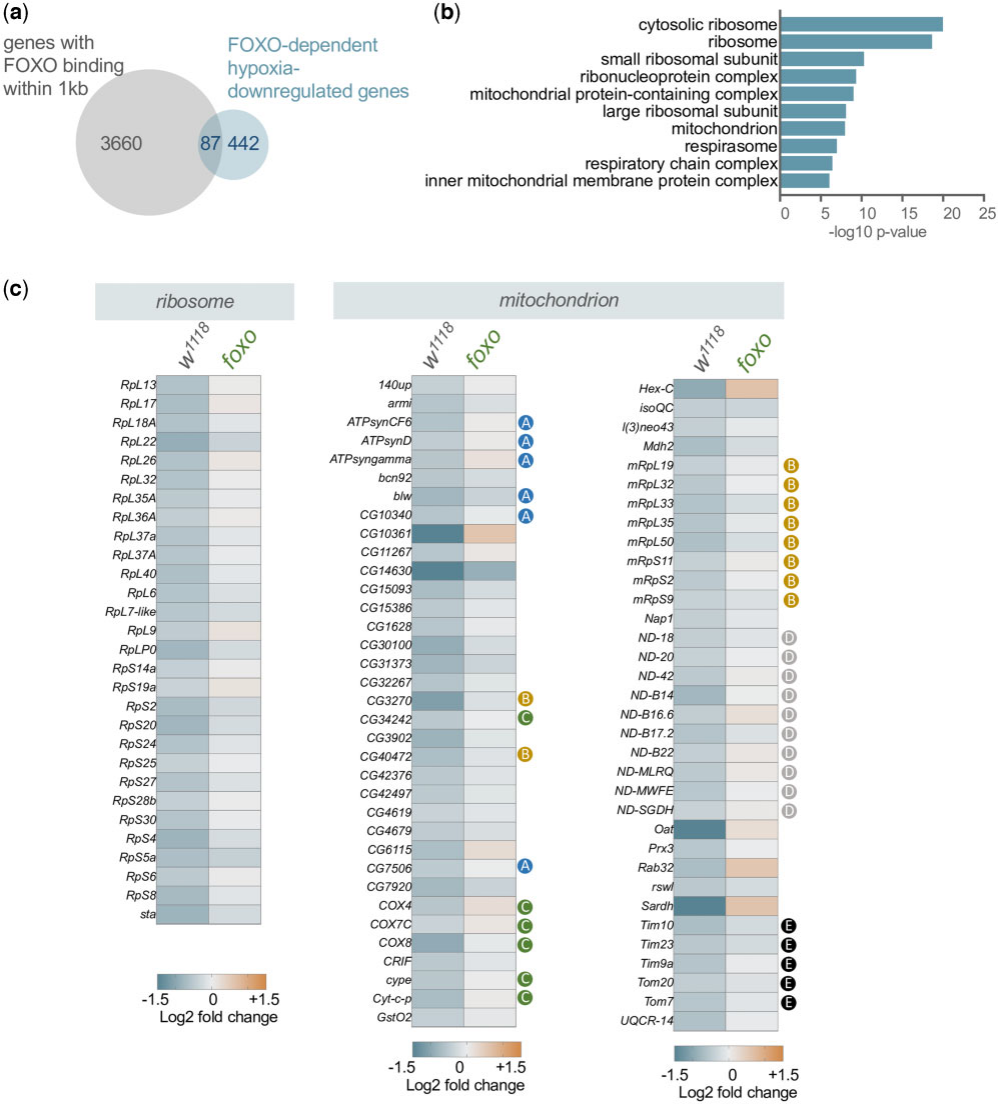
Hypoxia downregulation of ribosomal protein and mitochondrial regulator gene expression requires FOXO. a) Venn diagram showing overlap between genes previously shown to have FOXO binding within 1 kb, as detected by ChIP, and FOXO-dependent downregulated genes identified in the present study. b) GO analysis (cell component category) of FOXO-dependent hypoxia suppressed genes (genes showing a significant decrease in mRNA expression following hypoxia exposure in *w^1118^* but not *foxo* mutants). c) Heatmap depicting the decreases in mRNA expression (Log2-fold change, hypoxia vs normoxia) of ribosomal protein genes and mitochondrial regulator genes in *w^1118^* and *foxo* mutants. Colored circles indicate different classes of mitochondrial genes (blue (A): ATP synthase subunits; orange (B): mitochondrial ribosomal proteins; green (C): cytochrome *C* oxidase subunits; gray (D): complex I subunits; black (E): mitochondrial transporters).

We then examined which downregulated genes were dependent on FOXO, by identifying genes that were significantly downregulated (<1.3-fold) in *w^1118^* flies but not *foxo* mutants. We chose a lower fold change value because the significantly downregulated genes tended to be less affected than the upregulated genes. From this analysis, we identified 529 genes (39% of 1,343 total downregulated genes) (Supplementary Table 1). Of these, 87 were previously shown to be bound to FOXO (Alic *et al.* 2011; Birnbaum *et al.* 2019), suggesting that FOXO-mediated decreases in gene expression in hypoxia are largely indirect (Fig. 5a) (Supplementary Table 1). We used GO analysis to examine the functional categories of FOXO-dependent downregulated genes and identified strong enrichment in 2 main classes—ribosomal proteins and mitochondrial regulators (Fig. 5b). We saw that genes coding for ribosomal proteins for both the small and large subunits showed reduced expression in *w^1118^* but not *foxo* mutant animals (Fig. 5c). We also found that many mitochondrial genes required FOXO for their downregulation in hypoxia, including known or predicted mitochondrial ribosome proteins, cytochrome *C* oxidase subunits, complex I subunits, ATP synthases subunits, and mitochondrial transporters (Fig. 5c). These results suggest that an important role for FOXO in hypoxia is to suppress both mitochondrial and protein synthetic activities.

## Discussion

We previously showed that the FOXO transcription factor was required for hypoxia tolerance (Barretto *et al.* 2020). One focus of this current study was to identify which genes might be FOXO-regulated in hypoxia. Our results indicate that hypoxia exposure alters (±1.5-fold or greater) the transcript levels of ∼2,300 genes in our control (*w^1118^*) line, indicating a widespread modification of gene expression. To identify which gene expression changes are FOXO dependent, we chose to identify which genes had significantly altered expression in *w^1118^* but not *foxo* mutants. This analysis showed that ∼40% of hypoxia-regulated genes required FOXO. Furthermore, using data from previous genome-wide FOXO ChIP studies (Alic *et al.* 2011; Birnbaum *et al.* 2019), we saw that approximately half the FOXO-dependent upregulated genes were directly bound by FOXO. These results suggest that FOXO is needed for widespread transcriptional changes upon hypoxia. Since the previous ChIP analyses were carried out in normoxic flies, and we see that FOXO is induced in hypoxia (Barretto *et al.* 2020), it is possible that the number of FOXO-bound genes in hypoxia might be even higher, although Alic *et al.* showed that upon stress induction, FOXO tended to localize to the same genomic sites as in the absence of stress but with higher intensity. A previous report examining genome-wide changes in gene expression upon hypoxia exposure in larvae showed that HIF-1 alpha was required for just under half of the changes in gene expression and that the transcription factor estrogen-related receptor (ERR) was also important for mediating many of the effects of hypoxia on gene expression (Li *et al.* 2013). This study and our findings suggest that HIF-1 alpha, ERR, and FOXO may mediate many of the widespread changes in gene expression when flies are in low oxygen conditions. For example, we previously showed that FOXO was required for hypoxia tolerance in larvae (Barretto *et al.* 2020), suggesting that it may cooperate or work in parallel with HIF-1 alpha and/or ERR to regulate hypoxia-mediated changes in gene expression at this developmental stage. Interestingly, we also saw that ERR mRNA levels were significantly increased upon hypoxia in adults (1.48-fold), although this was below our cutoff of 1.5-fold. Nevertheless, this suggests that ERR may also be important for hypoxia-mediated gene expression changes in the adult.

Hypoxia-upregulated genes were enriched for kinases, regulators of small GTPases, and regulators of gene expression such as transcription factors and chromatin modifiers. This suggests that a major response to hypoxia is widespread alterations in cell–cell signaling pathways and their downstream transcriptional effectors. We found that upregulation of many of these signaling genes was dependent on FOXO and likely direct, since many of these bound FOXO. Interestingly, regulators of the Hippo pathway were among the FOXO-dependent upregulated genes. The Hippo pathway has been best studied in the context of cell growth and proliferation especially in epithelial, neural, and stem cells (Ma *et al.* 2019; Wu and Guan 2021). In these cells, the pathway often functions to couple cell-to-cell adhesion and cell polarity cues to the regulation of the downstream transcription factor Yorkie. Among the hypoxia-upregulated genes were several cell polarity/cell adhesion factors (*Ed*, *dlg1*, *sdt*, and *baz*) and signaling molecules (*hpo*, *mats*, *pez*, and *mts*) that function to negatively regulate Yorkie, suggesting that this may be an important regulator of hypoxia-mediated transcriptional responses. This regulation of Yorkie-mediated transcription may be important for regulation of stem or germ cell division upon hypoxia in adult flies. Yorkie can also regulate the processes of tracheal formation and immune signaling, which are both important in hypoxia. Recent studies have also shown that the mammalian homolog of Yorkie, Yap1, controls hypoxia-mediated angiogenesis in bone, suggesting that regulation of Hippo/Yorkie signaling may be a conserved hypoxia response (Sivaraj *et al.* 2020).

One kinase that showed FOXO-dependent increase in hypoxia was Hipk. We saw that this increase occurred across multiple tissues and was required for flies to survive hypoxia. These results point to Hipk as a regulator of hypoxia tolerance. As well as regulating Hippo/Yorkie signaling, Hipk can modulate other signaling pathways such as JNK, JAK/STAT, Wingless signaling (Lee, Swarup, *et al.* 2009; Huang *et al.* 2011; Chen and Verheyen 2012; Poon *et al.* 2012; Verheyen *et al.* 2012; Blaquiere *et al.* 2014; Tettweiler *et al.* 2019; Kinsey *et al.* 2021; Steinmetz *et al.* 2021), as well as Notch signaling (Lee, Andrews, *et al.* 2009), a pathway that we saw enriched in the KEGG analysis of hypoxia-upregulated genes. Thus, Hipk’s role in hypoxia tolerance may rely on regulation of any one of these pathways. Hipk has also been shown to induce glycolysis in larval epithelial tissues where it promotes tumor-like overgrowth (Wong *et al.* 2019). Hence, the hypoxia-mediated induction of Hipk may also be needed to induce glycolysis, a widely described metabolic response to low oxygen. Interestingly, a recent report showed that the *C. elegans* homolog of Hipk, hpk1, was needed for survival in low oxygen (Doering *et al.* 2022), suggesting a common role for Hipk in organismal hypoxia tolerance in both worms and flies.

Among the genes showing reduced expression in hypoxia, we saw strong enrichments for genes involved in egg production and translation. Furthermore, we saw that acute hypoxia exposure induced a transient (∼6 day) suppression of female fecundity and reduced translation and tRNA synthesis in both larvae and adults. Reproduction is an energetically costly process and therefore may be suppressed to ensure appropriate allocation of energetic resources to promote survival during periods of stress and stress recovery. The reduced overall fecundity we saw in hypoxia-exposed females may have occurred due to reallocation of energetic resources away from new egg production and egg-laying, or reduced provision of maternal sugar and lipid stores into new eggs leading to impaired development and decreased viability of progeny at either the embryonic or larval stages. These types of tradeoffs between fecundity and stress responses have been seen in *Drosophila* in response to other environmental challenges. For example, upon infection with bacteria, fungi or viruses, flies have been shown to reduce their reproductive output and capacity (Schwenke *et al.* 2016). Moreover, germline-deficient females that cannot produce eggs have enhanced immunity compared with fertile flies (Short *et al.* 2012). Similarly, nutrient starvation leads to reduced germline stem cell division and reduced egg production in females (Drummond-Barbosa and Spradling 2001; LaFever *et al.* 2010; Ables *et al.* 2012).

Protein synthesis is also an energetically costly process that has been estimated to account for at least one-third of a cell’s ATP use (Buttgereit and Brand 1995). Hence, it is not surprising that suppression of protein synthesis is a conserved response to hypoxia that is seen in many animals and that can promote hypoxia tolerance (Hofmann and Hand 1994; Hochachka *et al.* 1996; Liu, Cash, *et al.* 2006; van den Beucken *et al.* 2006; Anderson *et al.* 2009; Scott *et al.* 2013). Our transcriptomic analyses suggest that one way that hypoxia suppresses protein synthesis is by reducing the expression of ribosome protein genes via FOXO. We also saw that FOXO was required for hypoxia-mediated suppression of many mitochondrial genes, including mitochondrial ribosomal proteins, mitochondrial transporters, and regulators of oxidative phosphorylation, such as subunits of ATP synthase, cytochrome *C* oxidase, and complex I. These results suggest that FOXO may also contribute to hypoxia tolerance by limiting energetically costly metabolic processes. FOXO suppression of ribosome protein and mitochondrial genes has also been seen in muscle following nutrient starvation in *Drosophila* larvae (Teleman *et al.* 2008). Furthermore, a recent study showed that the FOXO homolog in *C. elegans*, daf-16, promotes a hypoxia tolerant phenotype by suppressing ribosomal protein gene expression and partially suppressing genes involved in oxidative phosphorylation (Hemphill *et al.* 2022). Hence, reducing both ribosome gene expression and mitochondrial oxidative phosphorylation may be common FOXO-mediated stress responses.

In conclusion, our transcriptome analysis supports a model in which FOXO promotes hypoxia tolerance through controlling the upregulation of cell signaling pathways while suppressing the energetically costly processes of protein synthesis and mitochondrial activity. Given the conserved roles for FOXO in mediating hypoxia tolerance in different animals (Scott *et al.* 2002; Mendenhall *et al.* 2006; Menuz *et al.* 2009; Liu *et al.* 2016; Barretto *et al.* 2020; Hemphill *et al.* 2022) and the alterations in FOXO transcription factor activity in diseases associated with hypoxia, such as cancer, stroke, and ischemia (Maiese *et al.* 2008; Fukunaga and Shioda 2009; Maiese *et al.* 2009; Liu *et al.* 2022), our findings highlight processes that may contribute to low oxygen adaptations in both normal and disease states.

## Supplementary Material

jkac263_Supplemental_Figures

jkac263_Supplementary_Table

jkac263_Supplementary_Material_Legends

## Data Availability

The RNA-sequence data have been deposited in NCBI’s Gene Expression Omnibus and are accessible through GEO Series accession number: GSE206206 (https://www.ncbi.nlm.nih.gov/geo/query/acc.cgi?acc=GSE206206). Summary data from the RNA-seq analyses are presented in Supplementary Table 1. Supplemental material is available at G3 online.
