# Supplementary material for: Transcriptome analysis of FOXO-dependent hypoxia gene expression identifies Hipk as a regulator of low oxygen tolerance in Drosophila
Source: G3 (Bethesda). 2022 Oct 6;12(12):jkac263. doi: 10.1093/g3journal/jkac263 (PMC9713431; doi:10.1093/g3journal/jkac263)
Supplement: jkac263_Supplemental_Figures [file jkac263_supplemental_figures.pdf]

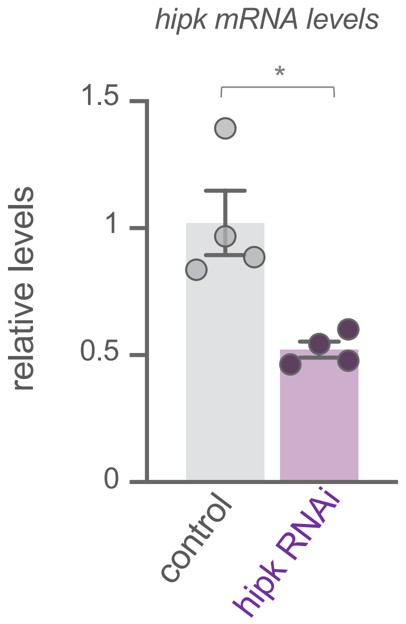

Supplemental Figure 1. Extent of RNAi-mediated knockdown of *hipk.*
qPCR analysis of *hipk* mRNA levels from control (*daGSG > hipk RNAi*, no RU486) vs *hipk* RNAi (*daGSG > Hipk RNAi*, RU486-treated) adult flies. Bars represent mean +/- SEM. Symbols represent individual data points, n=4 per condition. * p<0.05, Students t-test.

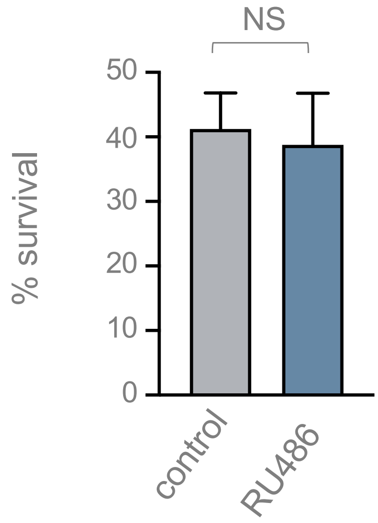

Supplemental Figure 2. RU486 treatment does not alter hypoxia survival. Control (*w^{1118}*) flies were fed food containing either control vehicle (ethanol) or RU486 (200mM) for 7 days and then survival following 20 hour hypoxia exposure was measured. Bars represent mean +/- SEM. n=7 groups of 20-25 flies per condition. NS = not significant, Students t-test.
